# Means, Motives and Opportunity: determinants of community health worker performance

**DOI:** 10.1136/bmjgh-2019-001790

**Published:** 2019-10-23

**Authors:** Aparna John, Thomas Newton-Lewis, Shuchi Srinivasan

**Affiliations:** 1 Institute of Development Studies, University of Sussex, Brighton, UK; 2 Oxford Policy Management, New Delhi, India

**Keywords:** health policy, health systems, health systems evaluation, public health

## Abstract

The performance of community health workers (CHWs) typically depends on the interaction between their motivation (their intent to achieve personal and organisational goals) and the constraints that they face in doing so. These constraints can be both at the individual level, for example, whether the worker has the skills and knowledge required to deliver on their job role, and the organisational level, for example, whether the worker is provided with the resources required to perform. Designing interventions to improve the performance of CHWs requires identifying the constraints to performance in a particular context. Existing frameworks on CHW performance tend to be derived empirically, identifying a broad range of intervention design and contextual factors that have been shown to influence CHW performance. These may not always be able to guide policy makers to identify the precise cause of a specific performance problem in a particular context and develop an appropriate policy response. This article presents a framework to help practitioners and researchers diagnose the constraints to performance of CHWs and guide programmatic and policy responses. The Means, Motives and Opportunity (MMO) framework has been adapted from the SaniFOAM framework used to identify the determinants of sanitation behaviours. It is based on three interdependent and interacting domains: means (whether an individual is capable of performing), motives (whether an individual wants to perform) and opportunity (whether the individual has the chance to perform). A wide range of data sources are expected to be used when applying the MMO framework, especially qualitative research that captures the perspectives and lived realities of CHWs and their communities. In this article, we demonstrate how the MMO framework can be applied to identify the constraints to CHW performance using the case study of Anganwadi Workers (village nutrition workers) in Bihar, India.

**Summary box**

- Addressing community health worker (CHW) performance challenges is acknowledged as essential to enable progress towards health-related goals.
- The Means, Motives and Opportunity (MMO) framework has been adapted from the SaniFOAM framework (used to diagnose the determinants of sanitation behaviours) to help practitioners and researchers to diagnose the constraints to performance of CHWs.
- The MMO framework is based on three interdependent and interacting domains: means (whether an individual is capable of performing), motives (whether an individual wants to perform) and opportunity (whether they individual has the chance to perform).
- A wide range of data sources are expected to be used when applying the MMO framework, especially qualitative research that captures the perspectives and lived realities of CHWs and their communities
- The MMO framework is applied to the case study of Anganwadi Workers (village nutrition workers) in Bihar, India. Results show that the main constraints to performance are system level constraints (opportunity), outside of the control of the worker.

## Introduction

Addressing community health worker (CHW) performance challenges is acknowledged as essential to enable progress towards health-related goals, including universal health coverage.^1^ CHWs serve as a link between the community and the public health system, delivering crucial outreach services to beneficiaries under community-based primary healthcare (CBPHC). Globally, CBPHC has the potential to avert 2.3 million deaths each year through increasing the coverage of evidence-based, maternal, newborn and child health interventions, far in excess of that which could be achieved through interventions at primary healthcare facilities and hospitals.^2^

The performance of CHWs typically depends on the interaction between their motivation (their intent to achieve personal and organisational goals) and the constraints that they face in doing so.^3^ These constraints can be both at the individual level, for example, whether the worker has the skills and knowledge required to deliver on their job role, and the organisational level, for example, whether the worker is provided with the resources required to perform. These factors interact: for example, a lack of resources can demotivate CHWs and can undermine the trust and respect that community members have in them. For example, in Ethiopia, CHWs who lacked supplies or equipment were sometimes seen as incompetent, and in Kenya beliefs that CHWs were withholding services created a sense of mistrust.^4^

Designing interventions to improve the performance of CHWs requires identifying the constraints to performance in a particular context. This requires a sensitive understanding of the motivations of the workers whose performance is being targeted and the environment in which they operate. Recent global research has shown that designing sustainable interventions needs to take into account the complexity of the communities that CHWs operate in (which are themselves complex adaptive systems)^5^ and the ‘intangible software’ of community health systems—the interests, power relations, norms, incentives and values impacting interactions between CHWs and community members and other parts of the health system.^6^ The literature emphasises how this complexity affects worker motivation and the level of trust in relationships between CHWs, their clients in communities and other actors in the health system; relationships which determine the performance of CHWs.^4^ For example, it has been found in India that when CHWs are used to facilitate community members to identify and overcome barriers to healthy behaviours and accessing health services through Participatory Learning and Action approaches, this strengthens relationships and enables improved performance of other services such as home visits.^7^ The complexity of communities may explain the differential impact of CHW home visits on antenatal care uptake and facility delivery among different social groups in India.^8^

Existing frameworks on CHW performance tend to be derived empirically, identifying a broad range of intervention design^9 10^ and contextual^11^ factors that have been shown to influence CHW performance. These may not always be able to guide policy makers to identify the precise cause of a specific performance problem in a particular context and develop an appropriate policy response. The ‘Means, Motives and Opportunity’ (MMO) framework presented in this paper seeks to provide a diagnostic framework that can identify the constraints to performance for a particular behaviour/task/service in a particular context, in order to guide programmatic and policy responses. It is adapted from the SaniFOAM framework developed for diagnosing the determinants of sanitation behaviours.^12^ It has been developed under the Frontline Worker Knowledge Synthesis grant received by Oxford Policy Management (OPM) from the Bill and Melinda Gates Foundation. The note also provides a case study to show how the framework has been applied to village nutrition workers—Anganwadi Workers (AWWs)—in Bihar, India.

## Means, Motives and Opportunity

**Figure 1:**
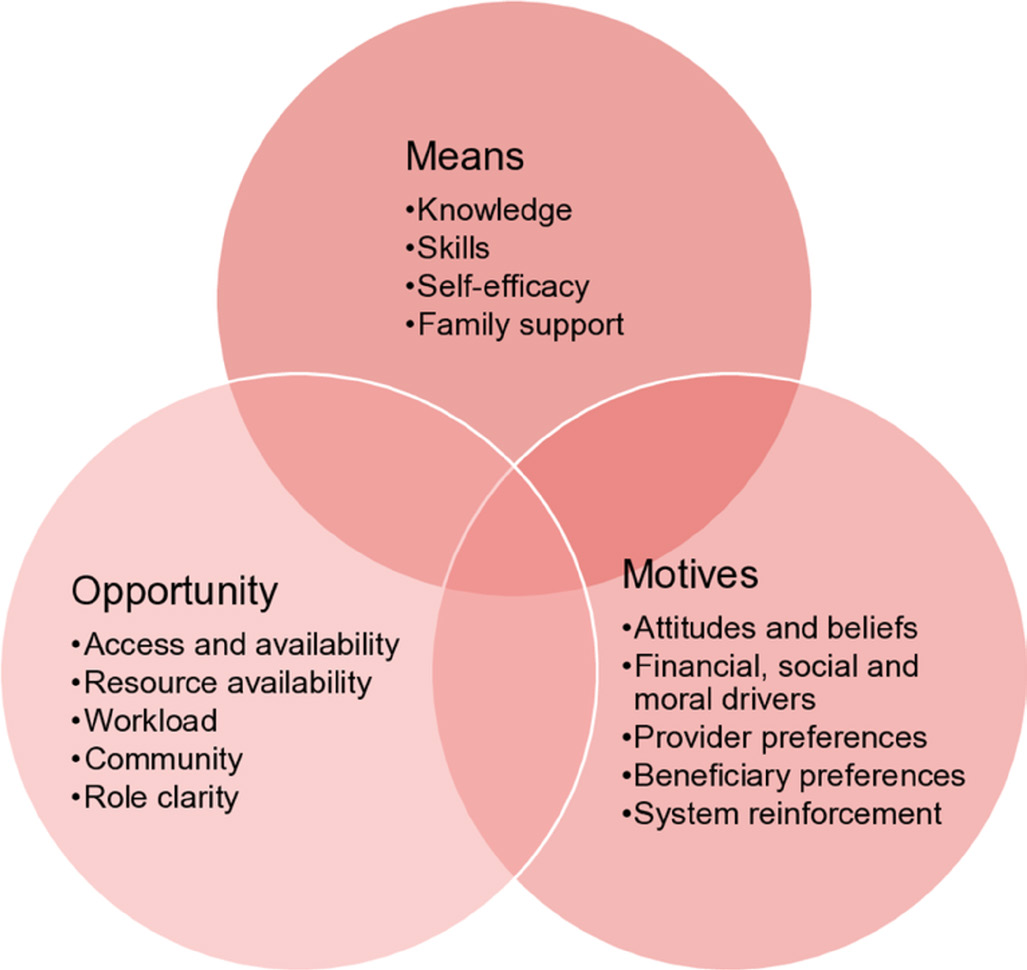
Means, Motives and Opportunity framework.

Building on the SaniFOAM framework, which itself uses a classification system commonly used in fields such as consumer behaviour, social marketing and organisational management, the MMO framework (figure 1) groups the determinants of performance into three interdependent and interacting domains:

- *Means:* Is the individual *capable* of performing the behaviour/task/service?
- *Motives:* Does the individual *want* to perform the behaviour/task/service?
- *Opportunity:* Does the individual have the *chance* to perform the behaviour/task/service?

These three areas are broken down into 14 determinants. These 14 determinants were developed based on a contextualisation from the SaniFOAM framework and comparative analysis with the existing frameworks on CHW performance,^6 9 11^ identifying factors related to performance constraints as opposed to intervention levers (such as training, incentives and supervision). Earlier versions of the framework were tested against emergent findings from ongoing research projects in India diagnosing the constraints to performance for different cadres of CHWs. This was used to assess the comprehensiveness and validity of the model and iterate it. It was also improved based on feedback when a draft was presented at a panel discussion on Optimising CHW performance in delivering RMNCH at the Women Deliver Conference, 2019.^13^

Means relates to the capability of the individual to perform the behaviour/task/service. It includes the following determinants:

- Knowledge: whether the individual has the requisite technical knowledge.
- Skills: whether the individual has the requisite competency (eg, communication skills, numeracy, problem solving, management).
- Self-efficacy: whether the individual has the self-belief in their ability.
- Family support: whether the individual has the required assistance from their family (eg, physical support, task assistance, informational support).

Motives relates to whether an individual wants to perform the behaviour/task/service. It includes the following determinants:

- Attitudes and beliefs: whether the individual believes that the behaviour/task/service is important (positive disposal leading to commitment) and is congruent with their remit.
- Financial, social and moral drivers: whether the individual believes that delivering the behaviour/task/service will help them fulfil their underlying motivations whether financial (eg, from incentives), social (eg, respect and positionality within the community) or moral (eg, a desire to achieve outcomes).
- Provider preferences: how important the individual believes the behaviour/task/service to be when compared with other work and non-work demands on their time.
- Beneficiary preferences: whether beneficiaries of the behaviour/task/service actively want it to be performed.
- System reinforcement: whether the individual is held accountable for performing the behaviour/task/service and receives positive reinforcement (eg, praise or reward) from managers, supervisors and peers for performing and negative reinforcement (eg, sanctions) for not performing.

Opportunity relates to whether the individual has the chance to perform the behaviour/task/service. It includes the following determinants:

- Access and availability: whether the platforms for undertaking the behaviour/task/service are available and beneficiaries are able to access them (eg, the facility is available, and beneficiaries can afford to attend it).
- Resource availability: whether the individual has the material (equipment, infrastructure, job-aids, supplies), financial, and human (helpers or other staff) inputs required.
- Workload: whether the overall workload on the individual is feasible.
- Community: whether the individual has the legitimacy to perform the behaviour/task/service and there are no other community barriers to delivery.
- Role clarity: whether the roles and responsibilities of the worker are clearly demarcated.

Applying the MMO framework requires the synthesis of different data types from a range of data sources. In particular, qualitative research that captures the perspectives and lived realities of CHWs and their communities is required. This is crucial to ensure that analysis and recommendations are based on a nuanced understanding of the complex dynamics of communities and the relationships that CHWs have with them (and other health system actors).

## Case study: Anganwadi Workers in Bihar

This case study demonstrates how the MMO framework can be applied to identify the constraints to performance of AWWs in Bihar, India. This case study was also undertaken in order to test and refine the MMO framework itself.

AWWs are village nutrition workers employed by India’s Integrated Child Development Services (ICDS) scheme. There are over 1.3 million AWWs employed across India, delivering a range of services including preschool education for children aged 3–6, the distribution of supplementary nutrition, the provision of health and nutrition counselling, the identification and referral and severely and acutely malnourished children and coordinating with village level health workers (Accredited Social Health Activists) and Auxiliary Nurse Midwives to organise Village Health, Sanitation and Nutrition Days (VHSNDs) where basic maternal and child health services such as antenatal care and immunisation are provided.^14^ Each AWW covers a population of approximately 1000.

The National Family Health Survey (NFHS-4) 2015–16 shows that, in Bihar, only 37% of pregnant women, 35% of breastfeeding women, 54% of children under 3 years and 43% of children aged between 3 and 6 receive any benefits from Anganwadi Centres (AWCs).^15 16^ This shows that a large proportion of the eligible population are completely excluded from the AWW service delivery net.

### Methods for case study

The MMO framework was applied to diagnose the causes of this low effective coverage. The analysis drew from a wide repository of available data from existing research on health system performance in the state of Bihar and secondary data from open sources. Framework analysis was used to synthesise insights and evidence from these and other sources against the MMO framework.

Data sources included a quantitative state representative survey of 380 AWWs in Bihar that measured their motivation, job satisfaction, personal characteristics, receipt of salary, the experience of supervision and management and other variables.^17^ The analysis also drew from facility-level data collected through a state representative survey of 380 AWCs in Bihar that measured their service availability and readiness, for example, the availability of infrastructure, funds, supplies, equipment.^18^ A qualitative study assessed the determinants of AWW performance in Bihar.^16^ This was part of a doctoral thesis submitted to the Institute of Development Studies, University of Sussex. It is based on 30 qualitative interviews with AWWs in Bihar conducted in 2015 undertaken for POSHAN (Partnerships and Opportunities to Strengthen and Harmonise Actions for Nutrition in India), led by IFPRI and funded by the Bill and Melinda Gates Foundation. This analysis also referred to secondary data sources to understand AWW performance^15^ and budgetary spending towards the ICDS programme.^19^

### Findings

A lack of means was found to be a barrier to performance. OPM’s survey data revealed that AWWs have limited levels of technical knowledge and only scored, on average, a third in a basic composite knowledge test.^17^ This has particular implications for services that require high degrees of knowledge such as nutrition counselling. Low levels of technical knowledge were in spite of a relatively high frequency of training, suggesting a low effectiveness of current training approaches. There is less existing evidence on whether AWWs have the broader skills, self-efficacy and social support required to deliver on their job responsibilities.

Motives (and motivation) were not found to be a binding constraint on performance. There is no evidence that AWWs did not expend the expected effort to provide services. AWCs were open and services made available as per mandated guidelines and AWWs adhered to processes that facilitate service delivery, such as creating due lists and undertaking eligibility surveys, within their constraints. There is no statistical correlation between motivation (self-reported, using a four point ordinal scale answer to the question ‘To what extent is the following statement true: overall, I’m motivated to work here’) and performance (measured through a composite index of services provided). AWWs are primarily motivated by the need to earn a salary to support their household income. Though many AWWs possess moral (to positively impact the community) and social (access to social and bureaucratic networks and prestige) motives, these are secondary to the financial motive. Delayed (and low) salaries are a major driver of job dissatisfaction. However, the need to retain their job ensures that they keep working and delivering even when their salaries are delayed. Delayed salaries also contributed to AWWs appropriating programme resources as a coping mechanism.

Within this overall narrative, AWWs were found to prioritise preschool teaching over other services, as it resonated with their self-identity as teachers, with teaching seen as a respectable, high status role in communities. They did not tend to self-identify as health and nutrition workers.

AWWs also were responsive to beneficiary preferences for product-oriented services such as the distribution of food over information-oriented services. This helps explain the lower coverage figures for health and nutrition counselling (18% of pregnant women according to NFHS 4) as compared with food distribution (33% of pregnant women^15^).

AWWs found supportive supervision (helping, mentoring and encouraging) very useful, presumably due to the knowledge gaps of AWWs. However, supervisors generally had knowledge levels no better than AWWs, limiting their effectiveness. Further, supervision in the system was mostly punitive and audit focused, which was demotivating and counterproductive.

The evidence suggest that opportunity determinants are the most binding on performance. In particular, supplementary nutrition allocations are only 44% of what is required for all eligible beneficiaries in an AWW’s catchment area (authors calculation), causing AWWs to restrict the number of beneficiaries who could receive it and reduce the amount received per beneficiary.

This has a disproportionate impact on service delivery because food acts as a hook to attract beneficiaries for other information-oriented services. Moreover, the necessary rationing of food-based services aggravates existing community tensions between different caste groups and due to seasonal migration, as all parts of the community perceived bias on the part of the AWW. This is further magnified by delays to fund disbursement and high levels of leakage within the system.

Resources are another major barrier to service delivery. Twenty per cent of AWCs are not operational and those that are have chronic deficiencies in infrastructure, equipment, supplies and registers.^17^ Additionally, large catchment areas and a lack of dedicated centres undermines accessibility and AWW safety and particularly affects services which require home visitations.

The analysis suggests that AWWs have limited agency over the factors that act as binding constraints to their performance as opposed to system level constraints (opportunity) such as availability of resources or individual level constraints (means) such as low technical knowledge. This may imply that an overall strategy should ideally focus on solving system level barriers to unlock performance and supporting AWWs to improve their ability. Without this, interventions solely targeting motives (such as incentives) may have limited impact.

The analysis reinforces how many of the MMO categories are interdependent. For example, the lack of supportive supervision meant that neither were the skills of AWWs improved (means), nor were the resource level constraints escalated and resolved (opportunities), and the punitive style of supervision was demotivating (motives). Furthermore, the lack of adequate resources provided to AWWs by the system undermined their motivation (motives) and their legitimacy in the community (opportunity). These interlinkages have implications for intervention design; interventions targeting one constraint may not unlock performance if other constraints remain binding. For example, it was found that providing AWWs with job aids (mobile phone based growth monitoring calculators) and financial incentives to undertake growth monitoring of children did not impact coverage due to limited community attendance at monthly VHSNDs where this was undertaken.^16^

## Conclusion

The MMO framework groups the determinants of CHW performance into three interdependent and interacting domains: means, motives and opportunity. This is further broken down into 14 determinants. The MMO framework provides an actionable framework for researchers and practitioners to diagnose the constraints to performance of CHWs in a particular context, taking into account their motivation and contextual constraints and can guide programmatic and policy responses. This complements existing frameworks on CHW performance which identify the broad range of factors that have been shown in the literature to influence CHW performance.

A wide range of data types and sources are expected to be synthesised in the diagnostic analysis using this framework. In particular, this should include qualitative research that captures the perspectives and lived realities of CHWs and their communities. The case study demonstrated how the MMO framework can be applied to examining the determinants of CHW performance using the example of AWWs in Bihar, India.
